# Atypical Presentation of Varicella-Zoster Virus Encephalitis in an Elderly Immunocompetent Adult: Early Diagnosis and Positive Outcome Following Treatment

**DOI:** 10.7759/cureus.88535

**Published:** 2025-07-22

**Authors:** Oluwaseun G Adewoye, Mohamed A Halim, Meena Srinivasan, Olasunkanmi H Owolabi

**Affiliations:** 1 Stroke Medicine, The Princess Royal Hospital, Shrewsbury and Telford Hospital NHS Trust, Telford, GBR; 2 Stroke Medicine, Nottingham University Hospitals NHS Trust, Nottingham, GBR; 3 Medicine, Shrewsbury and Telford Hospital NHS Trust, Telford, GBR

**Keywords:** acute encephalopathy, antiviral therapy, atypical presentation, csf pcr diagnosis, elderly immunocompetent, herpes zoster ophthalmicus, neurological recovery, stroke mimic, varicella zoster virus, vzv encephalitis

## Abstract

Varicella encephalitis following herpes zoster ophthalmicus (HZO) is a rare but serious complication that can occur due to the reactivation of the varicella-zoster virus (VZV). HZO involves the ophthalmic division of the trigeminal nerve, typically presenting with a dermatomal rash, and can lead to various ocular complications. In some cases, this reactivation can extend to the central nervous system, resulting in encephalitis, which can lead to significant morbidity and mortality, particularly in immunocompromised or elderly individuals. This report describes a case of VZV encephalitis complicating HZO in an elderly immunocompetent male who presented with altered mental status following an initial presentation of HZO in the absence of a dermatomal rash, with a tentative diagnosis of an acute ischemic stroke. Early diagnostic confirmation was achieved through cerebrospinal fluid analysis and polymerase chain reaction, which identified VZV central nervous system infection. The patient was administered intravenous acyclovir, leading to complete neurological recovery. This case underscores the necessity of including VZV encephalitis in the differential diagnosis of acute encephalopathy, even when typical dermatological signs are absent. Furthermore, it emphasizes the critical role of prompt antiviral therapy in ensuring favorable clinical outcomes. Additionally, this case illustrates that VZV encephalitis can mimic stroke-like symptoms, highlighting the potential for misdiagnosis and the importance of considering infectious etiologies in such presentations.

## Introduction

Varicella-zoster virus (VZV) is a neurotropic alpha herpesvirus that causes varicella (chickenpox) during primary infection and can reactivate later in life as herpes zoster (shingles). Globally, varicella affects approximately 140 million people annually, with 4.2 million severe complications requiring hospitalization, predominantly in unvaccinated children under five in temperate regions and adults in tropical areas [1]. Herpes zoster ophthalmicus (HZO), occurring in 10-20% of shingles cases, demonstrates rising incidence (3.6% annually) in the United States, particularly among adults aged >60 years and immunocompromised individuals [2,3]. Neurological complications like VZV encephalitis, though rare (1/30,000-50,000 varicella cases), carry mortality rates of 5-15% in immunocompetent patients and up to 80% in immunocompromised populations, with 30-60% of survivors experiencing long-term sequelae [3,4].

In the United Kingdom, herpes zoster affects 11.5% of adults in their lifetime, with higher incidence in women (12.6% vs. 10.3% men), white ethnic groups, and those with digestive disorders [5]. While pediatric varicella encephalitis hospitalizations declined by 72% post vaccination, UK adults aged >65 face elevated encephalitis risks (4.32-5.23 cases/100,000/year), with VZV implicated in 5% of cases [4].

Although VZV reactivation typically presents with a dermatomal rash, it can lead to severe neurological complications, including encephalitis, particularly in immunocompromised individuals. However, VZV encephalitis in immunocompetent patients remains rare and diagnostically challenging due to atypical presentations such as absence of a rash in up to 30% of cases [4]. Early diagnosis and treatment are critical, as delayed intervention heightens morbidity and mortality [6].

Our case report describes an elderly immunocompetent patient with VZV encephalitis complicating HZO who presented without a rash and was successfully treated with acyclovir.

## Case presentation

We present a case of an 80-year-old gentleman with a medical history of ischemic heart disease, squamous cell carcinoma of the right ear (cured four years prior), actinic keratosis, hypertension, transient ischemic attack, and dyslipidemia.

He presented to the emergency department with acute confusion and agitation. The symptoms developed suddenly while returning from an eye clinic, where he had received topical ganciclovir two weeks earlier as treatment for right eye HZO anterior uveitis. Paramedics reported left-sided weakness and slurred speech, prompting hospital admission.

On examination, the patient was afebrile (temperature: 36.7°C) with stable vital signs: blood pressure of 148/78 mmHg, heart rate of 77 beats per minute, and oxygen saturation of 99% on room air. Neurological assessment revealed a Glasgow Coma Scale (GCS) score of 14/15 (E:4, V:4, M:6). The right pupil was dilated due to recent mydriatic eye drop administration, but no focal neurological deficits were observed. Notably, he had no dermatomal rash. The National Institutes of Health Stroke Scale (NIHSS) score was 1, attributed to confusion.

**Figure 1:**
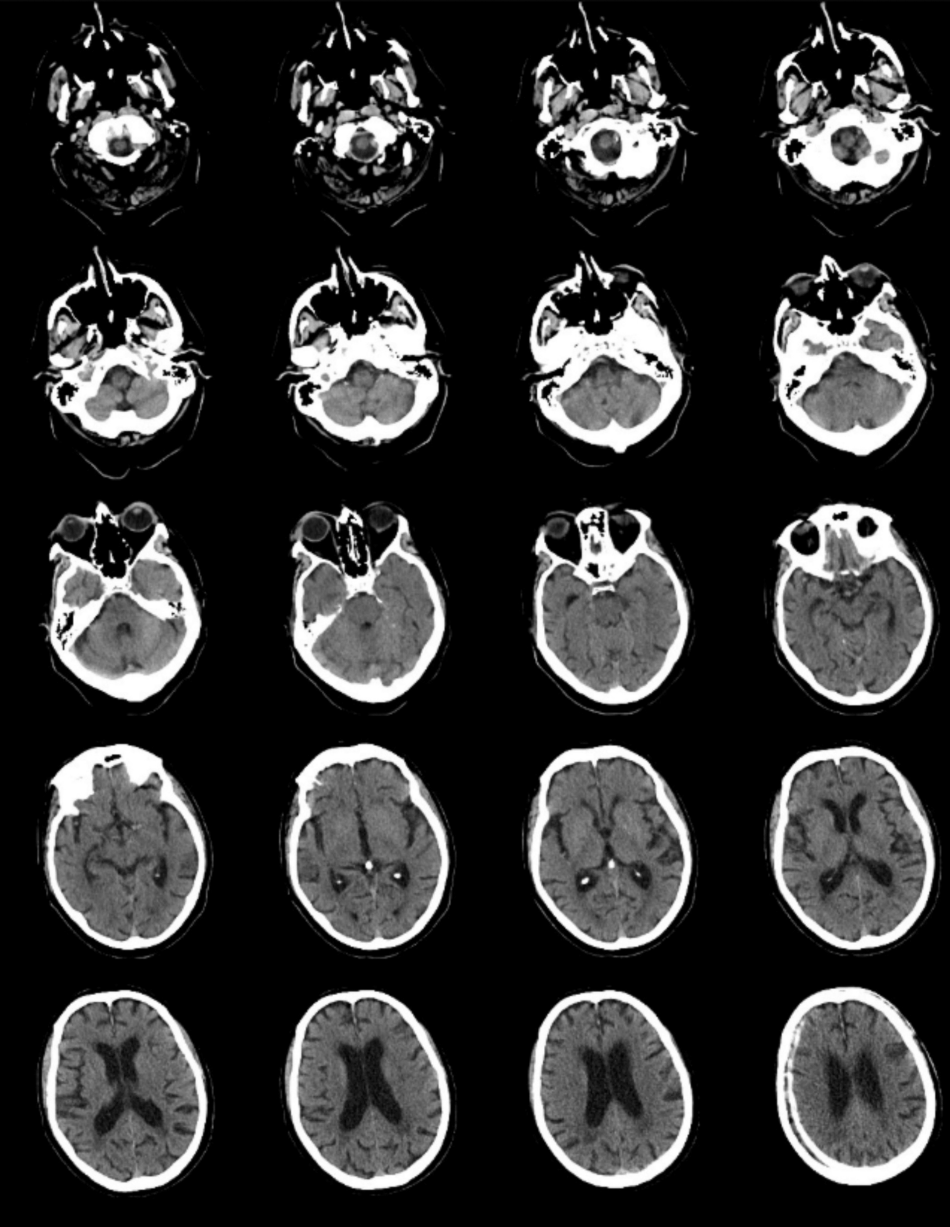
Axial sections of unenhanced computed tomography (CT) of the head showing no acute pathology.

Initial non-contrast computed tomography (CT) of the head (Figure 1) showed no acute abnormalities. Laboratory investigations showed normal inflammatory markers: C-reactive protein (CRP) of 2, white cell count of 9.2 (10^9^/L) (neutrophils: 7.2, lymphocytes: 1.4), and hemoglobin and platelets were within normal limits at 150g/l and 214 (10^9^/L), respectively. Renal, liver, and thyroid function tests, serum bone profile, vitamin B12, and folic acid levels were all within normal limits. Given the clinical suspicion of viral encephalitis, empirical treatment with intravenous acyclovir (750 mg three times daily) was initiated. Cerebrospinal fluid (CSF) analysis (Table 1) showed elevated CSF protein (1435 mg/L), normal glucose (5.7 mmol/L), and a mononuclear pleocytosis (86 x 10^6/L, 100% mononucleocytes). CSF polymerase chain reaction (PCR) confirmed VZV infection, while herpes simplex virus (HSV) PCR was negative. CSF cultures showed no bacterial growth.

**Table 1:** CSF analysis showing VZV positivity, CSF mononucleocytosis, significantly elevated CSF protein, and mild hyperglycorrhachia. ** Depicts abnormal CSF findings. CSF: cerebrospinal fluid; VZV: varicella-zoster virus; PCR: polymerase chain reaction.

Test name	Patients results	Normal range
CSF protein	1435 mg/L**	150-450 mg/L
CSF glucose	5.7 mmol/L**	2.7-4.44 mmol/L
CSF WBC	86 x10^6l (100% mononucleocytes)**	0-5^6/L
CSF RBC	0	0
CSF viral PCR	Herpes simplex virus: negative; varicella-zoster virus: positive**	

The patient made full neurological improvement within 48 hours of commencing intravenous antiviral therapy, with complete resolution of confusion, GCS improving to 15/15, and no evidence of residual neurological deficits. He completed a full 14-day course of parenteral acyclovir without complications and was subsequently discharged in stable condition. At the three-month follow-up, he remained in satisfactory general health and was pending further evaluation by ophthalmology.

## Discussion

VZV encephalitis represents an uncommon but severe neurological sequela of VZV reactivation, occurring in 0.1-0.2% of herpes zoster cases, with heightened risk in disseminated infections affecting dermatomes proximate to the central nervous system (CNS) [6,7]. The pathophysiology entails retrograde viral transmission from latently infected sensory ganglia of the trigeminal nerve to the CNS, inciting inflammatory responses that manifest as headaches, altered mental status, seizures, or focal deficits [8]. While classic presentations combine HZO rash with neurological symptoms, up to 30% of immunocompetent patients exhibit atypical features, such as rash absence, complicating timely diagnosis [9,10]. In this case, the patient’s prior HZO anterior uveitis history prompted rapid CSF analysis and PCR testing, circumventing diagnostic delays.

CSF profiling in VZV encephalitis typically reveals lymphocytic pleocytosis, elevated protein, and normal glucose levels [11,12], consistent with our patient's CSF findings. PCR detection of VZV DNA in CSF remains the diagnostic gold standard due to its high sensitivity (95-100%) and specificity [13]. Early intravenous acyclovir administration, a viral DNA polymerase inhibitor, is critical, as delayed treatment correlates with prolonged hospitalization and residual deficits. This patient’s full recovery underscores the efficacy of prompt antiviral initiation.

In addition to antiviral therapy, supportive care is essential in managing symptoms and complications associated with varicella encephalitis. Maintaining adequate hydration and nutrition is vital to support overall health and assist in recovery. Medications may be prescribed to alleviate symptoms such as pain and fever [14]. Regular neurological monitoring allows for the early detection of complications such as seizures and changes in consciousness, which are common in encephalitis patients. This early detection is crucial for timely intervention and management, potentially preventing long-term neurological damage [15,16].

Complications of VZV encephalitis include cerebrovascular ischemia and hemorrhage, venous thrombosis, and chronic neurocognitive impairment [17]. Mortality and morbidity risks escalate with advanced age, immunosuppression, comorbidities (e.g., diabetes), and severe baseline neurological impairment (e.g., GCS ≤8) [18].

## Conclusions

This case report underscores the diagnostic challenges of VZV encephalitis in an immunocompetent elderly patient with prior HZO who presented without a dermatomal rash. Early recognition through CSF analysis and PCR testing, coupled with prompt acyclovir therapy, was pivotal in achieving a favorable outcome, highlighting the imperative for clinicians to maintain high suspicion for VZV encephalitis in cases of acute encephalopathy even in the absence of cutaneous manifestations to optimize therapeutic efficacy and reduce morbidity.
